# Using CBCT for pretreatment range check in proton therapy: a phantom study for prostate treatment by anterior–posterior beam

**DOI:** 10.1120/jacmp.v16i6.5212

**Published:** 2015-11-08

**Authors:** El Hassane Bentefour, Stefan Both, Shikui Tang, Hsiao‐Ming Lu

**Affiliations:** ^1^ Advanced Technology Group Ion Beam Applications s.a., Louvain‐la‐Neuve Belgium EU; ^2^ Department of Radiation Oncology University of Pennsylvania Philadelphia PA USA; ^3^ Department of Radiation Oncology Massachusetts General Hospital Boston MA USA

**Keywords:** CBCT, WEPL calculation, range uncertainty, proton therapy

## Abstract

This study explores the potential of cone‐beam computed tomography (CBCT) for monitoring relative beam range variations due to daily changes in patient anatomy for prostate treatment by anterior proton beams. CBCT was used to image an anthropomorphic pelvic phantom, in eight sessions on eight different days. In each session, the phantom was scanned twice, first at a standard position as determined by the room lasers, and then after it was shifted by 10 mm translation randomly along one of the X, Y, or Z directions. The filling of the phantom bladder with water was not refreshed from day to day, inducing gradual change of the water‐equivalent path length (WEPL) across the bladder. MIMvista (MIM) software was used to perform image registration and re‐alignment of all the scans with the scan from the first session. The XiO treatment planning system was used to perform data analysis. It was found that, although the Hounsfield unit numbers in CBCT have substantially larger fluctuations than those in diagnostic CT, CBCT datasets taken for daily patient positioning could potentially be used to monitor changes in patient anatomy. The reproducibility of the WEPL, computed using CBCT along anterior–posterior (AP) paths across and around the phantom prostate, over a volume of 360 cc, is sufficient for detecting daily WEPL variations that are equal to or larger than 3 mm. This result also applies to CBCT scans of the phantom after it is randomly shifted from the treatment position by 10 mm. limiting the interest to WEPL variation over a specific path within the same CBCT slice, one can detect WEPL variation smaller than 1 mm. That is the case when using CBCT for tracking daily change of the WEPL across the phantom bladder that was induced by spontaneous change in the bladder filling due to evaporation. In summary, the phantom study suggests that CBCT can be used for monitoring day to day WEPL variations in a patient. The method can detect WEPL variation equal to or greater than 3 mm. The study calls for further investigation using the CBCT data from real patients. If confirmed with real patients' data, CBCT could become, in addition to patient setup, a standard tool for proton therapy pretreatment beam range check.

PACS number: 87.55.Tm

## I. INTRODUCTION

Cone‐beam CT (CBCT) is standard for image‐guided radiation therapy (IGRT) in photon clinics.^(1)^ Its potential in proton therapy treatment has also been investigated,^(2)^ although the proposed usage is limited only to geometric targeting (i.e., beam‐target alignment). An interesting question is, whether CBCT can be used also for tracking daily variations of the patient water‐equivalent path length and, subsequently, could be used as tool for beam range check before proton therapy treatment?^(3)^

Range uncertainty is one of the most difficult problems in particle therapy treatment.^4^, ^5^ Such uncertainty translates directly into potential undershooting or overshooting of the Bragg peak in the patient. Neither situation is desirable in treatment because either the distal part of the tumor is missed (undershooting) or healthy tissues immediately behind the tumor receiving an unacceptable dose (overshooting). Sources of range uncertainty occur both in treatment planning and in treatment delivery. In the former, uncertainties in the Hounsfield unit (HU) numbers of the treatment planning CT and their conversion to proton stopping power ratios can translate into uncertainties in the calculation of the water‐equivalent path length (WEPL), and in turn the prescribed beam range.^(6)^ In treatment delivery, daily variation of patient's anatomical configuration and setup could change the WEPL along the beam path for the particular treatment fraction, potentially causing the overshooting or undershooting, clearly more so for certain types of treatment (e.g., prostate) than for others (e.g., intracranial tumors), because of the deeper range and more heterogeneous beam path.

To date, only few techniques for *in vivo* range verification have been tested, but are not used in a clinical routine. Positron emission tomography (PET) imaging is a post‐treatment that is based on treatment‐activated isotopes.^7^, ^8^, ^9^ While that method indeed provides a verification of the treatment, the drawback is that it verifies the beam range only after the treatment, but not before, since the imaging depends on isotope activities produced by the treatment itself. It cannot be applied to treatments where daily variations in patient anatomy and setup may require that the beam range be adjusted prior to each treatment fraction. Proton radiography has been suggested and tested for *in vivo* range verification,^(10)^ but so far it has never been integrated into a clinical setup. And, recently, a new method has been suggested, prostate treatment by anterior or anterior oblique field, which may significantly improve rectal sparing by using the much sharper distal dose falloff rather than the currently employed parallel opposed field approach.^(11)^ Such treatment would require range verification prior to each beam delivery session to guarantee that the beam is covering the full prostate but stopping before the rectum. This range verification can be done by the method newly suggested by Lu^(12)^ and further developed by Gottschalk et al.^(13)^ This method uses a simple yet robust relationship between the timing of the treatment beam energy modulation and the WEPL of the beam in the patient. Further, it has been demonstrated by Bentefour et al.^(14)^ that this method can measure the proton beam range in heterogonous media and in anthropomorphic pelvic phantom with better than 2 mm accuracy. A clinical version of an *in vivo* range verification system using this method with a 12‐detector array of 1 mm diodes mounted on a rectal balloon is under development.^15^, ^16^

Undoubtedly, CBCT has its limitations when compared to the standard CT.^(17)^ Moreover, it cannot help reduce range uncertainties coming from the treatment planning system nor from the treatment delivery system. Its usefulness for range uncertainties lies in its potential for providing patient positioning data that can be used for daily monitoring of the WEPL between the patient's skin and the treatment target. Such information, if accurate, can be used to detect unexpected changes in the patient WEPL that, if not detected, would worsen the beam range uncertainty and subsequently the quality of the treatment. It is obvious that using CBCT for detecting any relative changes in patient WEPL along the treatment beam path does not enable direct verification of the proton beam range; however, it can be used to indirectly derive the beam range correction for this particular treatment session. That is because one can quantify the needed range correction by relating the measured relative changes in the WEPL computed using CBCT data to the already base‐lined WEPL from previous treatment sessions where the beam range was actually verified by the PET method^7^, ^9^ or other *in vivo* range verification techniques like time resolved dosimetry.^12^, ^13^, ^14^, ^15^

The patient WEPL between two points of interest along the beam path can be computed using CBCT patient positioning data, but it is unknown with what precision, and whether it can be accurate and reproducible enough to actually be clinically useful. Many factors, such as heterogeneities in the beam path and the organs' interplay, could influence the CBCT performance; however, it is certain that the intrinsic reproducibility of the CBCT imaging from day to day and its sensitivity to setup errors is a major factor because knowing the patient WEPL is a necessary condition to be met before proceeding with any further steps. In this study, we investigate the intrinsic imaging variability of the CBCT and its effect on the precision of the WEPL calculations. We address the specific case of using the CBCT setup data to monitor the WEPL of an anthropomorphic pelvic phantom along the beam path for prostate treatment with anterior‐posterior field. In this configuration, WEPL variations could result from changes in the body surface due to daily inconsistencies in positioning setup, as well as from changes in the pelvic phantom bladder filling from day to day.

## II. MATERIALS AND METHODS

### A. CBCT experiment

A custom anthropomorphic phantom for the pelvic region (from The Phantom Laboratory, Salem, NY) was used in this study. The phantom was designed specifically for dose verification measurements related to prostate treatment. Using such an anthropomorphic phantom was crucial, as it offers relatively realistic distribution of tissue densities: its bladder can be filled with water and it has a rectal cavity that can house a rectal water balloon, as widely used for internal immobilization during prostate treatment. Real human pelvic bones were used in constructing the phantom. Samples of the tissue‐equivalent materials that were used respectively for soft tissue and prostate organs were CT‐scanned and their respective densities were verified to be near water equivalent. The respective ratios of their geometric thicknesses to their water‐equivalent thickness were found equal to 1.02 and 1.04.

The pelvic anthropomorphic phantom was scanned in eight sessions on eight different days, simulating eight treatment fractions. The CBCT scans were performed using a Varian On‐Board Imager (OBI) (Varian Medical Systems, Palo Alto, CA) version 1.4 CBCT machine with settings of 125 kV, 80 mA, and 20 msec. The CBCT scans were intentionally performed on different days in order to capture the effects of any variations in the performance of the imaging equipment and the effects of positioning errors resulting from user errors. All the CBCT scans were done at CBCT field view of 450 mm and slice thickness of 2.5 mm. The pelvic phantom was also CT‐scanned on Day One of the experiment. This CT scan is used to determine the absolute WEPL value, which was then used to baseline the WEPL values calculated from the CBCT of the same day, which was then used as reference in the monitoring of the daily WEPL variation in comparison with the WEPL measured using CBCT data from Day Two to Day Eight.

In each session, the phantom was first aligned to the room lasers using predefined marks at the surface of the phantom, which put the phantom prostate at the treatment room isocenter. Then, a water‐filled rectal balloon was placed in the phantom rectal cavity. The phantom was scanned twice: first at the treatment position as determined by the room lasers, and then after it was shifted 10 mm by simple translation along a randomly chosen axis (x, y, or z) on any given day. The purpose of the second scan was to simulate an actual patient setup procedure where the patient was not yet positioned correctly for treatment when CBCT was employed. It is important to evaluate the effect of this shift on CBCT accuracy for the WEPL calculations because CBCT is generally used only once per treatment session, when the patient is in the process of being aligned to the treatment position.

**Figure 1:**
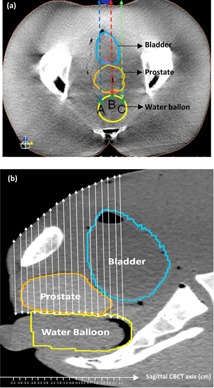
CBCT transverse view (a) showing the various paths (A, B, and C) along which the WEPL was calculated, and CBCT sagittal (b) illustrating the distribution of these paths along the pelvic phantom rectal wall.

Figure 1(a) shows a transversal CBCT slice with representation of three AP paths (A, B, and C) along which the WEPL was calculated. Figure 1(b) shows a sagittal view of a CBCT slice of the pelvic phantom showing all paths B. All paths together, A, B, and C, cover a volume of 60 mm along axial direction (x‐axis), by 40 mm along the lateral direction (y‐axis), by the geometric thickness of the phantom, which varies with the shape of the phantom abdomen along the axial direction. The beginning and end points of each of all paths (A, B, C) along all CBCT slices, were manually determined.

In order to measure the ability of CBCT for capturing systematic change in the WEPL of the pelvic phantom, we devised the following strategy. On the first day of the experiment, before the first CBCT scan, we filled the bladder of the pelvic phantom with water to full level. Then, no water was added to it for the following seven days of the experiment. This allowed the slow evaporation of the water to create a gradual change in the water‐equivalent thickness of the phantom across the bladder. This change was quantified by measuring the geometric thickness of the water level in the bladder from day to day, to establish the ground truth, then comparing that with the computed WEPL using the CBCT datasets.

### B. Data analysis

Although during each imaging session the phantom was aligned to the room lasers as accurately as possible, it became evident from the initial analysis that it is difficult to match the corresponding geometric locations across all the scans. For this reason, we performed image registration based on the phantom bony anatomy using MIM software (MIM Software Inc., Cleveland, OH), which is based on a constrained, intensity‐based, free‐form deformable registration. Then we realigned all CBCT scans with the scan from the first session (chosen as the default reference). Then, the resampled CBCT scans were used for the analysis.

The XiO treatment planning system (Elekta Instrument AB, Stockholm, Sweden) was used to perform the analysis. This system has a feature that allows for density‐to‐Hounsfield units (HU) calibration, which is performed by entering HU values for air and water obtained by sampling regions in the image with known tissue densities. The Hounsfield unit numbers are then converted to relative stopping power, which is used to compute the WEPL between any two points of interest within the pelvic phantom. XiO was also used to measure the daily change of the water level in the bladder by measuring the geometrical thickness between the tip of the bladder and the level of the water along the AP direction.

For each CBCT dataset, we sampled the HU numbers of water over a selected volume of water in the bladder of the phantom. This selected volume, equal to 48 cc and about 26% of the total volume of the bladder (184 cc), was contoured in the middle of the bladder far from any of its edges and was kept identical for all CBCT datasets. The mean values and the standard deviation of the HU distribution were then calculated from each of the CBCT scans in order to make a basic evaluation of the daily reproducibility of the CBCT scans. (The CBCT scan of Day Three with the random shift of the phantom is missing from the analysis because, by mistake, it was not acquired.)

Then, we performed the WEPL calculation along the AP treatment beam path, as in the case of prostate treatment by a straight anterior field. This specific field was selected as a useful case for evaluating the sensitivity of CBCT computed WEPL to changes in the patient radiological thickness. The WEPL values were calculated between the anterior surface of the phantom and the inner surface of the rectal wall, which was visually identified. The calculations were performed along three different paths over 33 consecutive axial CBCT slices. The selected paths were laterally spaced by roughly 20 mm; the CBCT slices were axially spaced by 2.5 mm. Some of the selected AP paths pass through pubic bones around the pubic symphysis and some of these paths pass through the inferior part of the bladder. All together, they cover the entire region of the prostate in addition to 15 mm of margins both superiorly and inferiorly.

The same WEPL calculations were performed for each of the resampled CBCT datasets. The daily variations in the WEPL values from all CBCT scans with the phantom at the room isocenter were calculated relative to the WEPL values measured from the CBCT of the first day. For CBCT scans with the phantom randomly shifted out of the room isocenter, the WEPL variations were calculated relative to the WEPL from CBCT scans acquired on the same day with the phantom at the room isocenter.

## III. RESULTS

### A. Intrinsic reproducibility of CBCT for water HU measurement

**Figure 2:**
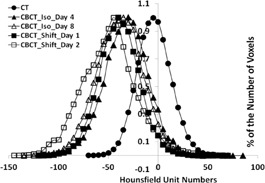
Distributions of HU number of water from the phantom bladder obtained by CT and CBCT scans. Only CBCT HU distributions with the lowest and highest mean HU numbers from the scans with phantom at isocenter and off isocenter are presented.

Figure 2 shows five distributions of the HU numbers of the water from the phantom bladder. It also shows, for reference, the distribution of water HU numbers measured using diagnostic CT (solid circles). The two distributions represented by solid triangles and open triangles correspond, respectively, to the lowest and highest HU distributions from the CBCT scans with the phantom alignment at the room isocenter. The two distributions represented by solid squares and open squares correspond, respectively, to the water HU distributions with the lowest and highest HU mean value from the CBCT scans of the phantom after it was randomly shifted by 10 mm from the room isocenter.

Table 1 shows, respectively, the mean and standard deviation (STD) of the distributions of the water HU numbers in the phantom bladder for all CBCT data sets. CT scans taken on Day One and on Day Eight of the study show that the distribution of the HU numbers from water in the phantom bladder was highly reproducible with a mean equal to −2 and an STD equal to 11. For the CBCT scans with the phantom at the room isocenter, the mean of the water HU values varied from −29 to −39 with the average at −33.4; the STD of the corresponding HU distributions varied between 21 and 32 with an average of 24.1. For the CBCT series where the phantom was shifted off the room isocenter by 10 mm, the HU mean value has a slightly larger variation over the seven sessions as it varies from −32 to −47 with the average at −39.6. The corresponding STD values roughly remained at similar magnitude as when the phantom was aligned at the room isocenter. In order to translate the above findings into the impact on CBCT WEPL reproducibility, we investigated the effect of the average error on the mean of the CBCT water HU distribution, which is ±9 HU, considering the worst case by combining data from CBCT scans with and without the phantom random shift by 10 mm, on the two‐point WEPL‐equivalent calibration that we have used in this study. The analysis shows that a difference of 10 HU in the water density calibration corresponds to a change of 1% on the WEPL. That is equivalent of a systematic error of 1.5 mm on the WEPL in our test case with range of approximately 150 mm.

**Table 1:** The mean and sigma of the distributions of the HU numbers of the water in the phantom bladder for all CBCT scans performed over the eight days with phantom at room isocenter and after it is shifted by 10 mm from the isocenter

		*Day 1*	*Day 2*	*Day 3*	*Day 4*	*Day 5*	*Day 6*	*Day 7*	*Day 8*	*Average*
CBCT with Phantom at room isocenter	mean	‐32	‐32	‐31	‐29	‐37	‐37	‐30	‐39	‐33.4
	sigma	32	20	22	21	23	23	28	24	24.1
CBCT with Phantom 10 mm of room isocenter	mean	−32	−47	—	−40	−42	−40	−38	−38	−39.6
	sigma	20	28	—	23	22	26	23	25	23.9

### B. Verification of CBCT potential as a QA tool for range uncertainty

#### B.1. Daily reproducibility of CBCT computed WEPL

Figures 3.1(a), 3.1(b), and 3.1(c) give the absolute WEPL values computed using the CBCT datasets, along paths A, B, and C, respectively. Figure 3.2(a), Fig. 3.2(b), and Fig. 3.2(c) give the actual variation of WEPL values from CBCT scans from Day Two to Day Seven relative to the WEPL values from the CBCT scan of Day One, which is used as the reference.

The absolute WEPL values, shown in Fig. 3.1(a), Fig. 3.1(b), and Fig. 3.1(c) increase as the slice position moves from inferior to superior. This is consistent with the slope of the anterior surface of the phantom as shown by sagittal view in Fig. 1(b). For slice positions inferior to 5 mm, 92% of the daily changes are less than 2 mm in magnitude and 70% of them are less than 1 mm, with the values from Day Eight as outliers for no apparent reason, particularly visible in Fig. 3.2(a). These statistics are for the changes in WEPL values over all paths (A, B, and C) relative to those of Day One.

For slice positions superior to 5 mm, the changes are much larger for paths A and B, and increased monotonically from Day Two to Day Eight. This is as expected, since these two paths pass through the bladder, as indicated in Fig. 1(a). The WEPL should decrease as the water in the bladder, as expected, slowly evaporates, as mentioned in the Materials and Methods section above. Path C, on the other hand, never passes through the empty part of the bladder; therefore, the daily changes in its WEPL have similar magnitude over all slice positions.

**Figure 3:**
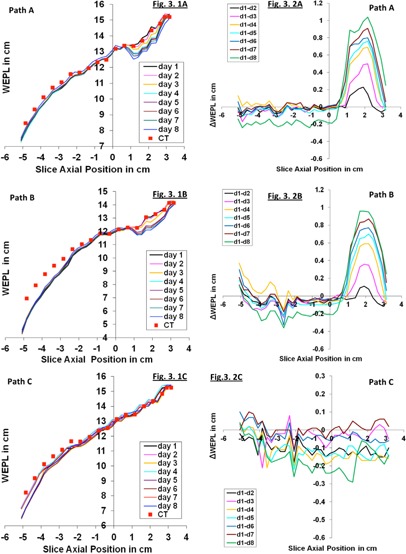
(1a), (1b), and (1c) give absolute WEPL from CBCT scans of phantom at isocenter over eight consecutive days: (2a), (2b), and (2c) show the corresponding WEPL variation.

#### B.2. Sensitivity of CBCT computed WEPL to error in phantom positioning

**Figure 4:**
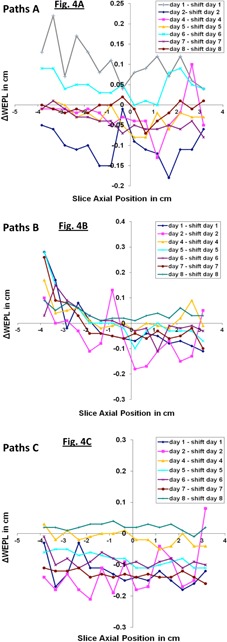
WEPL variation from CBCT scans taken the same day with the phantom at room isocenter and displaced by 10 mm off the room isocenter. The same measurements are repeated for three different AP paths (A, B, and C) and over eight consecutive days.

Figure 4 shows the WEPL variations calculated along the three paths A, B, and C across the pelvic phantom when an error of 10 mm is made in the phantom position. The WEPL variations were calculated from each of the two CBCT scans taken on the same day, with the phantom at the room isocenter and after it was shifted by 10 mm. The magnitude of the obtained WEPL variations over all the paths A, B, and C is within ± 2 mm for about 98% of the individual path and within ± 1 mm for 75% of the individual path. This result is comparable to the measured daily WEPL variations, shown in Fig. 3, for the path outside the phantom bladder. This indicates that one can use CBCT datasets from slightly misaligned patients (with an error up to ± 10 mm) for computing the WEPL with similar confidence as when using CBCT datasets from well‐aligned patients.

#### B.3. Sensitivity of CBCT to systematic change in the WEPL

The sensitivity of the CBCT to systematic changes in the phantom WEPL was evaluated by comparing the daily changes of the WEPL calculated using CBCT datasets to the absolute WEPL change measured by taking advantage of the spontaneous water evaporation from the phantom bladder over the eight days of the experiment.

**Figure 5:**
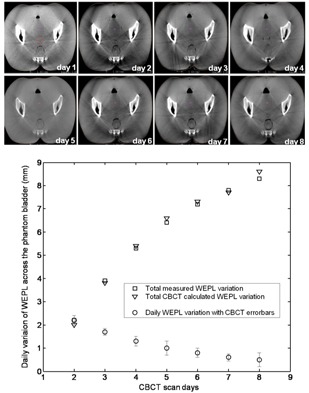
The top panel visualizes the change of the water level in the phantom bladder over the course of eight days. The bottom panel shows the agreement of CBCT computed WEPL (triangles) with measurement of the change in the water thickness (ground truth) along the same path.

The top panel of Fig. 5 shows the same CBCT slice over the eight days of the experiment, visualizing the daily change of the water level in the phantom bladder. The bottom panel of Fig. 5 reports the measurements of the physical change in the water thickness in the phantom bladder along the path B (open triangles) and compares it to the changes in WEPL across the same path calculated using CBCT data sets (open squares). The actual daily decrease of the WEPL is represented in the same figure by the plot with the open‐circle symbol, the error bars representing the accuracy of the WEPL calculated using CBCT datasets.

The physical measurement of the water level in the bladder shows systematic decrease over the period of the experiment, with an average of 1.2 mm per day. A rapid decrease (2 mm per day) is seen over the first days of the experiment and slower decrease (0.5 mm per day) for the last days of the experiment. This change in the decay rate of the water level in the bladder is a false symptom; the reality is that the water evaporation rate is fairly constant from day to day, but it appears to be decreasing from day to day due to the widening of the phantom bladder as one moves posteriorly. This can be verified by normalizing the change in height of the water column to the change in the air volume in the bladder.

## IV. DISCUSSION

We conducted a phantom study to evaluate the HU uncertainties in CBCT scans used for routine patient setup and their effects on WEPL calculations. Although the proposed use of CBCT should apply to all cancer tumor sites, we specifically have studied, as a first step, the case of prostate cancer treatment by anterior proton beams. The choice of this test‐case may not be a good illustration of today's clinical practice in proton therapy because proton treatment of the prostate with AP field and AP oblique field is not common.^(18)^ However, the choice was based, on the one hand, on convenience: the pelvic phantom allows for testing the ability of CBCT to track systematic changes in the WEPL by varying the water level in the bladder. On the other hand, it is expected that the treatment of prostate with AP field will eventually become more and more common as proton therapy practice inches towards intensity‐modulated proton treatment (IMPT), within which treating prostate with AP straight and AP oblique fields could become regular practice.

With positive results, this study could establish the basis for further exploration of the potential role of CBCT in managing the interfractional component of beam range uncertainty. If CBCT is demonstrated to be useful for beam range monitoring, it will specifically address the uncertainties in the beam range caused by changes in the patient anatomy during the course of the treatment, such as in the cases when the patient gains or losses weight, or when the patient's cavities' filling changes or when the tumor size diminishes due to treatment. CBCT won't be of any use for addressing range uncertainties that originate from the conversion of HU number into relative stopping power or from proton therapy equipment‐related uncertainties.

In reference to our experiment, one should highlight the following limitations, which, if overcome, would likely improve the outcome of this study. 1) We used CBCT settings (KVp, mA, pulse duration in ms) that are primarily optimized for geometric setup and low dose to the patient. Thus, it is likely that optimizing these settings could improve HU accuracy in tissue density, while delivering acceptable doses to the patient. 2) For convenience, we have used the simple two‐point WEPL calibration method that is available in the XiO treatment planning system. It is likely that using a more robust method for calibrating HU number to relative stopping power, such as the stochiometric method, could further improve the results of this study. 3) Other potential improvement of the results of this study may come from using a more recent CBCT system that offers higher quality in the CBCT imaging. 4) We manually determined the end point for WEPL measurement (points at the rectal wall). The reproducibility of this manual operation from one CBCT slice to another cannot be perfect and therefore could be a source of error. Although such error is small, it is part of the overall reproducibility of the CBCT that we obtained. An automated, robust algorithm could be used to determine the rectal wall with better accuracy and so could improve the overall reproducibility of the CBCT‐based WEPL calculation.

In contrast to the above possible improvements on the experiment side and thus of the result of the study, one should highlight other elements that could limit the potential of the method. 1) Possible artifacts in CBCTs, that could rise in case of real patients due to patient movement, or streak artifacts because of bowel movements and gas, which can impact the WEPL calculation accuracy. 2) The likelihood of increased sensitivity of the CBCT data to geometric corrections in the case of CBCT mounted on a proton therapy gantry due to its larger mechanical design in comparison to a standard linac‐mounted CBCT. Despite the above limitations, the results of this study should remain valid for a CBCT system mounted on a proton therapy gantry, providing that its imaging quality is identical to or better than that used in this study.

In a nutshell, our results shows that the WEPL values calculated from CBCT are smaller than those from CT, as shown in Fig. 3. This is not surprising, given the known limitations of CBCT for capturing tissue density. But, in term of reproducibility of the WEPL measurement using CBCT data, it is promising that the CBCT HU distributions appears to be sufficient for detecting discrepancy in WEPL values between CBCT scans from different days, allowing therefore the detection of any accumulation of systematic change or abrupt change in the patient WEPL. This conclusion applies both to cases when the patient setup is complete and when it is incomplete (i.e., in the case of a phantom shifted by 10 mm off room isocenter). In numbers, our results shown in Fig. 3(b) and Fig. 4 can be translated as follows: the maximum random variations in WEPL computed across multiple slices of CBCT scan that cover approximately a volume of 360 cc, is 3 mm for 97% of the measured data points from all eight days of the study. If one considers only the WEPL variation from any two days, then the maximum WEPL variation is only 1 mm for 97% of the data points, which suggests the potential of using CBCT to detect systematic WEPL change as small as 1 mm, as observed in Fig. 5.

Translating our result to today clinical practice is encouraging. It is widely accepted in today's proton therapy practice that, due to uncertainties in CT calibration and the conversion of HU numbers to relative stopping power (RSP), one has to account for a systematic error on the beam range up to 4%.^(6)^ Therefore, in order to guarantee full coverage of the tumor, a margin, typically 3% to 5%, depending on the proton therapy institution, is added to the range of the treatment beam estimated by the treatment system.^(5)^ If one considers that reducing this error margin by 50% is clinically relevant, which we believe it is, and, that given the fact that 150 mm, which requires a 6 mm error margin, is a reasonable average of beam ranges needed to treat deep‐seated tumors, then a minimum of 3 mm (i.e., 50% of 6 mm) reproducibility of WEPL measurement is needed for any method that monitors range uncertainty to be clinically useful. This criterion is clearly met in our study, which evaluates the sensitivity of CBCT for WEPL monitoring by studying its reproducibility for WEPL calculation. As result, we believe that CBCT has the potential for being used as tool to monitor day‐to‐day changes in the patient's anatomy, which could help avoid dramatic range errors in the treatment beam. These encouraging preliminary results invite further studies to fully explore the potential of this method and its suitability for use in clinical practice.

In an envisioned workflow, the patient will receive the CBCT for positioning. The CBCT will be manually or automatically registered to the treatment planning CT to obtain the position corrections (translation and rotation), the accuracy of this registration to be investigated in respect of the nature of the registration methodology and in light of uncertainties related to prostate position due to interplay.^(18)^

Such registration can be achieved automatically or manually, either by fiducial markers, or by soft tissue anatomy. CBCT generally has lower contrast in the pelvic region, but the edge of the rectal water balloon can be contoured with reasonable certainty most of time. (Radiopaque contrast may be added to the water used to fill the water balloon to help with identifying the balloon edge.) The contours will form a surface along the inside of the rectal wall. The registered CBCT data can then be sampled based on the position correction in the coordinates of the treatment planning CT. For every point on this surface, a WEPL value can be calculated from the body surface along the beam path. The obtained WEPL values form a 2D WEPL map in the beam's eye view. When this map is compared to maps obtained from prior treatment fractions, discrepancies can be evaluated and the beam range can be adjusted accordingly. By comparing the WEPL from the actual treatment session to the WEPL from the first day of treatment one can verify if the total cumulated WEPL changes during the course of the treatment have become unacceptable for continuing use of the same treatment plan. The effectiveness of this approach should be first validated against an independent technique for range verification such as prompt gamma camera measurement,^19^, ^20^ the PET method,^(7^–^9)^ proton radiography,^(10)^ or the *in vivo* time resolved dose rate method.^11^, ^12^, ^13^, ^14^, ^15^

## V. CONCLUSIONS

We performed a phantom study to evaluate the intrinsic uncertainty in WEPL calculations based on CBCT scans used for routine patient positioning. It was found that, although the Hounsfield unit numbers in CBCT have substantially larger fluctuations than those in diagnostic CT, CBCT could detect, in a phantom study, daily or cumulative variations of WEPL equal or greater than 3 mm within the treatment volume. The results call for further studies based on CBCT data for real patients and suggest that CBCT could potentially be used as a QA tool to monitor on a daily basis the changes in patient anatomy along the axes of the treatment fields. Such potential could be exploited to avoid dramatic errors in the range of the treatment beam, and would reveal when patient replanning is needed.
